# Reconfigured metabolism brain network in asymptomatic microtubule-associated protein tau mutation carriers: a graph theoretical analysis

**DOI:** 10.1186/s13195-022-01000-z

**Published:** 2022-04-11

**Authors:** Li Liu, Min Chu, Binbin Nie, Lin Liu, Kexin Xie, Yue Cui, Yu Kong, Zhongyun Chen, Haitian Nan, Kewei Chen, Pedro Rosa-Neto, Liyong Wu

**Affiliations:** 1 Department of Neurology, Xuanwu Hospital, Capital Medical University, Changchun Street 45, Beijing, China; 2 Department of Neurology, Shenyang Fifth People Hospital, Shenyang, China; 3 Beijing Engineering Research Center of Radiographic Techniques and Equipment, Institute of High Energy Physics, Chinese Academy of Sciences, Beijing, China; 4 School of Nuclear Science and Technology, University of Chinese Academy of Sciences, Beijing, China; 5 Department of Neurology, Second Hospital of Shanxi Medical University, Taiyuan, China; 6 Banner Alzheimer’s Institute, University of Arizona, School of Mathematics and Statistics, Arizona Alzheimer’s Consortium, Arizona State University, Tempe, USA; 7 McGill Centre for Studies in Aging, Alzheimer’s Disease Research Unit, Montreal, H4H 1R3 Canada; 8 National Clinical Research Center for Geriatric Diseases, Beijing, China

**Keywords:** Frontotemporal dementia, MAPT gene, FDG-PET, Brain network, Graph theory

## Abstract

**Background:**

Studies exploring topological properties of the metabolic network during the presymptomatic stage of genetic frontotemporal dementia (FTD) are scarce. However, such knowledge is important for understanding brain function and disease pathogenesis. Therefore, we aimed to explore FTD-specific patterns of metabolism topology reconfiguration in microtubule-associated protein tau (MAPT) mutation carriers before the onset of symptoms.

**Methods:**

Six asymptomatic carriers of the MAPT P301L mutation were compared with 12 non-carriers who all belonged to the same family of FTD. For comparison, we included 32 behavioral variant FTD (bvFTD) patients and 33 unrelated healthy controls. Each participant underwent neuropsychological assessments, genetic testing, and a hybrid positron emission tomography (PET)/magnetic resonance imaging (MRI) scan. Voxel-wise gray matter volumes and standardized uptake value ratios were calculated and compared for structural MRI and fluorodeoxyglucose (FDG)-PET, separately. The sparse inverse covariance estimation method (SICE) was applied to topological properties and metabolic connectomes of brain functional networks derived from ^18^F-FDG PET/MRI data. Independent component analysis was used to explore the metabolic connectivity of the salience (SN) and default mode networks (DMN).

**Results:**

The asymptomatic MAPT carriers performed normal global parameters of the metabolism network, whereas bvFTD patients did not. However, we revealed lost hubs in the ventromedial prefrontal, orbitofrontal, and anterior cingulate cortices and reconfigured hubs in the anterior insula, precuneus, and posterior cingulate cortex in asymptomatic carriers compared with non-carriers, which overlapped with the comparisons between bvFTD patients and controls. Similarly, significant differences in local parameters of these nodes were present between asymptomatic carriers and non-carriers. The reduction in the connectivity of lost hub regions and the enhancement of connectivity between reconfigured hubs and components of the frontal cortex were marked during the asymptomatic stage. Metabolic connectivity within the SN and DMN was enhanced in asymptomatic carriers compared with non-mutation carriers but reduced in bvFTD patients relative to controls.

**Conclusions:**

Our findings showed that metabolism topology reconfiguration, characterized by the earliest involvement of medial prefrontal areas and active compensation in task-related regions, was present in the presymptomatic phase of genetic FTD with MAPT mutation, which may be used as an imaging biomarker of increased risk of FTD.

**Supplementary Information:**

The online version contains supplementary material available at 10.1186/s13195-022-01000-z.

## Background

Frontotemporal dementia (FTD) is a clinically, genetically, and pathologically heterogeneous group of syndromes characterized by the degeneration of the frontal and temporal cortices [1]. Up to 40% of FTD cases are genetic, with the most common cause being mutations of the microtubule-associated protein tau (MAPT) gene, especially in China. FTD is characterized by extensive tau pathology, and the most prevalent clinical phenotype is the behavioral variant of FTD (bvFTD) [2, 3]. Increasing evidence has confirmed the presence of pathophysiological changes during the presymptomatic stage of genetic FTD [4]. Families with a presence of MAPT mutations provide an ideal model to investigate brain structure and function during the presymptomatic stage and investigate the pathogenesis of FTD.

Over the last decade, several studies have reported the gray matter atrophy and white matter integrity loss in asymptomatic MAPT mutation carriers, although these changes are not common [5]. It has been proposed that functional changes precede the occurrence of atrophy and thus may constitute one of the earliest features of neurodegeneration [6]. The only two studies using ^18^F-fluorodeoxyglucose (FDG)-positron emission tomography (PET) have revealed local hypometabolism in presymptomatic MAPT mutation carriers, which is thought to reflect neuronal dysfunction [7, 8]. However, increasingly, studies have advocated that brain function is not solely attributable to the nature of isolated regions or subnetworks connections. Rather, it emerges from the overall network organization of the brain as a whole, that is, the systematic balance of connectomes encompassing integration and segregation functions [9]. To date, limited MRI studies in asymptomatic mutation carriers of genetic FTD families focused on regional functional connectivity, which has not allowed an understanding of how networks are embedded and interact within the complex brain system [6, 10]. Metabolic network topology based on FDG-PET is considered a useful measure that reflects neuronal communication signaling at the network and whole-brain levels, and it can capture neural and synaptic activity more directly than can functional magnetic resonance imaging (fMRI) [11]. However, the overall organization of the whole-brain network in presymptomatic MAPT mutation carriers remains poorly understood, which could help to elucidate the pathogenesis and allow monitoring of the earliest changes associated with FTD.

In the current study, we assessed the changes in the topological properties and connectome integrity in asymptomatic carriers and non-carriers of the same pedigree using graph theoretical analysis of ^18^F-FDG PET/MRI data. We aimed to explore the specific patterns of metabolism topology reconfiguration in MAPT mutation carriers before the onset of symptoms. We predicted that the local disruption of metabolic network topology would be present in asymptomatic MAPT subjects, whereas topology reconfiguration would maintain an efficient organization of the brain’s functional network.

## Methods

### Subjects

Eighteen asymptomatic participants were recruited from a family with an autosomal dominant P301L mutation in the MAPT gene through the frontotemporal lobar degeneration database, which was established at the Department of Neurology of Xuanwu Hospital, China. All participants underwent genetic screening, and six participants were found to be carriers of the mutation, and 12 were mutation-negative, who were used as controls in this study. Each participant underwent clinical interviews, physical examinations, neuropsychological assessments, and cerebral ^18^F-FDG PET/MRI. All subjects had been followed up prospectively with annual clinical examinations between September 2017 and October 2021 at Xuanwu Hospital. During the 4-year follow-up period, all subjects remained symptom-free, and none of them has developed any bvFTD symptoms or any other neurodegenerative disease.

We also identified 32 subjects seen at the Department of Neurology of Xuanwu Hospital, China, who fulfilled the 2011 consensus probable bvFTD criteria [12]. All of these patients also underwent clinical interviews, physical examinations, neuropsychological assessments, genetic testing, and cerebral ^18^F-FDG-PET/MRI. Of these 32 bvFTD patients, five had MAPT mutations (one with a P301L mutation [c.1907C>T], one with a V337M mutation [c.2014G>A], one with the N296N mutation [c.1839T>C], one with the R5C mutation [c.13C>T], and one with the D54N mutation [c.160G>A]). In addition, a total of 33 participants from the database of individuals with normal cognition, which was established at the Department of Neurology of Xuanwu Hospital during the same period, were enrolled as controls. Each participant underwent the same neuropsychological assessments as the patients. The demographics of the subjects are shown in Table 1.

**Table 1:** Demographic and neuropsychiatric assessment data

	Asymptomatic MAPT carriers (*n* = 6)	Non-carriers in family (*n* = 12)	BvFTD patients (*n* = 32)	Controls (*n* =33)	*p* value^†^, MAPT carriers vs non-carriers	*p* value*, bvFTD patients vs controls
Age	49.00 ± 3.90	42.25 ± 9.206	59.13 ± 9.97	54.27 ± 11.17	0.19	0.07
Sex (male/female)	3/3	7/5	16/16	15/18	0.99	0.81
Years of education	8.67 ± 0.52	10.55 ± 3.80	10.79 ± 4.64	11.23 ± 3.35	0.25	0.69
MMSE	28.67 ± 0.82	28.36 ± 2.06	12.54 ± 7.01	28.47 ± 2.17	0.92	< 0.0001
MoCA	26.50 ± 1.23	26.18 ± 3.06	9.323 ± 6.25	26.13 ± 3.40	0.68	< 0.0001
AVLT: immediate recall	23.83 ± 2.32	26.00 ± 7.32	8.15 ± 7.04	22.40 ± 5.25	0.94	< 0.0001
AVLT: delayed recall	8.67 ± 2.25	9.27 ± 3.16	1.74 ± 2.68	8.43 ± 3.48	0.71	< 0.0001
BNT	25.00 ± 1.00	24.90 ± 2.08	10.78 ± 6.52	25.38 ± 3.48	0.97	< 0.0001
CDR	0 ± 0	0 ± 0	1.76 ± 1.22	0 ± 0	–	< 0.0001
NPI-Q	0 (0–6)	0 (0–1)	24.13 ± 21.03	1.29 ± 4.53	0.89	0.0003
FBI	0 (0–6)	0 (0–2)	23.78 ± 14.39	1.667 ± 4.082	0.84	0.001
MBI-C	0 (0–14.25)	0 (0–1)	19.82 ± 15.25	2.20 ± 6.96	0.68	0.002

Data are presented as means ± standard deviation or medians (Q1–Q3)

*AVLT*, auditory verbal learning test; *BNT*, Boston Naming Test; *CDR*, Clinical Dementia Rating; *FBI*, Frontal Behavioral Inventory; *MBI-C*, Mild Behavioral Impairment Checklist; *MMSE*, Mini-Mental State Examination; *MoCA*, Montreal Cognitive Assessment; *NPI-Q*, Neuropsychiatry Inventory Questionnaire

^†^Two-sided *p*-values for continuous variables refer to the Mann-Whitney tests, and two-sided *p*-values for categorical variables refer to Pearson’s chi-squared test

*Two-sided *p*-values for continuous variables refer to unpaired *t*-tests, and two-sided *p*-values for categorical variables refer to Pearson’s chi-squared test

### Neuropsychological assessments

The neuropsychological test battery consisted of widely used neuropsychological assessments that measure the cognitive function in the domains of memory, language, and behavioral abnormality. Global cognitive screening measures included the Mini-mental State Examination (MMSE), the Montreal Cognitive Assessment (MoCA), and the Clinical Dementia Rating (CDR) Scale. Word list memory was evaluated using Rey’s Auditory-Verbal Learning Test (AVLT). Language was measured using the Boston Naming Test (BNT). The severity of behavioral abnormality was assessed using the Frontal Behavior Inventory (FBI), the Neuropsychiatry Inventory Questionnaire (NPI-Q), and the Mild Behavioral Impairment Checklist (MBI-C).

### PET/MRI acquisition parameters

All images were acquired on a hybrid 3.0 T time-of-flight PET/MRI scanner (SIGNA PET/MR, GE Healthcare, WI, USA) [13]. PET and MRI data were acquired simultaneously using a vendor-supplied 19-channel head and neck union coil. Subjects were injected intravenously with l8F-FDG (3.7 MBq/kg) and underwent three-dimensional (3D) T1-weighted sagittal imaging and 18F-FDG-PET imaging 40 min later during the same session.

A 3D T1-weighted fast field echo sequence (repetition time [TR] = 6.9 ms, echo time [TE] = 2.98 ms, flip angle = 12°, inversion time = 450 ms, matrix size = 256 × 256, field of view = 256 × 256 mm^2^, slice thickness = 1 mm, 192 sagittal slices with no gap, voxel size = 1 × 1 × 1 mm^3^, and acquisition time = 4 min 48 s) was used for data acquisition. Static ^18^F-FDG-PET data were acquired using the scanning parameters of matrix size = 192 × 192, field of view = 350 × 350 mm^2^, and pixel size = 1.82 × 1.82 × 2.78 mm^3^ and included corrections for random coincidences, dead time, scatter, and photon attenuation. Attenuation correction was performed according to the brain MRI (atlas-based coregistration of two-point Dixon) [14]. The default attenuation correction sequence was automatically prescribed and acquired as follows: LAVA-Flex (GE Healthcare) axial acquisition, TR = 4 ms, TE = 1.7 ms, slice thickness = 5.2 mm with a 2.6-mm overlap, 120 slices, pixel size = 1.95 × 2.93 mm, and acquisition time = 18 s.

### Structural image preprocessing

Data were preprocessed using the Computational Anatomy Toolbox (CAT12) toolbox segment data pipeline implemented within Statistical Parametric Mapping 12 (SPM12, www.fil.ion.ucl.ac.uk/spm). Structural MRI images were normalized to standard Montreal Neurological Institute (MNI) space using diffeomorphic anatomical registration through exponentiated lie algebra normalization as implemented in SPM12. The images were then smoothed using an 8-mm full-width half-maximum isotropic Gaussian kernel for all directions.

### PET image preprocessing

PET images were preprocessed using SPM12, implemented in MATLAB (MathWorks, Natick, MA). After normalization of the structural MRI images, the transformation parameters determined by the T1-weighted image spatial normalization were applied to the co-registered PET images for PET spatial normalization. The images were then smoothed using an 8-mm full-width half-maximum Gaussian kernel for all directions. Finally, PET scan intensity normalization to the mean of the cerebellar gray matter was applied to create standardized uptake value ratio (SUVR) images.

### Voxel-based analysis for structural MRI and FDG-PET

The preprocessed structural and ^18^F-FDG-PET SUVR image data were used to perform voxel-wise whole-brain comparisons between asymptomatic mutation carriers and non-carriers, the bvFTD patients and controls.

### Whole-brain metabolic connectome: sparse inverse covariance estimation analysis

We used sparse inverse covariance estimation (SICE), which is a method previously validated by Huang et al. [15]. A series of nodes (*N* = 172) that represent the brain regions of interest (ROIs) for the connectivity analysis were selected to cover the whole brain [16, 17]. The ^18^F-FDG-PET signal was extracted from each ROI in each subject to obtain subject × ROI matrices. The SICE algorithm was then applied to these matrices to generate metabolic connectivity matrices. Graph theory measures were computed from the metabolic connectivity matrices, and a bootstrapping procedure was performed to test for differences between the groups.

The most important nodes of the network (i.e., the hubs) were identified by selecting nodes with a participation coefficient that was one standard deviation higher than the mean degree centrality.

### Default mode and salience networks: independent component analysis

Spatial independent component analysis (ICA) of the preprocessed FDG-PET images was performed using the GIFT toolbox (http://mialab.mrn.org/software/). Imaging data from each subject were arranged in a sequence and whitened and reduced using principal component analysis. Then, an Infomax ICA algorithm, which minimizes mutual information, was used to estimate the independent spatial components [18]. The GIFT software was used to estimate the optimal number of components, which were based on the assessment of the entropy rate of independent and identically distributed Gaussian random processes [19]. To display the voxels that contributed most to the resulting spatial maps, voxel intensity values were converted into *z*-scores, and image values were visualized using a threshold of > 1.96 [18]. Finally, components of the default mode networks and salience networks were identified according to anatomical information [18].

### Statistical analyses

The GraphPad Prism software (version 8.3.0, GraphPad Software Inc., La Jolla, CA) was used to evaluate the statistical significance. Numerical variables are presented as means ± standard deviations or medians and ranges, depending on the normality of the distribution. Comparative analyses of numerical variables were performed using non-parametric Mann-Whitney tests between asymptomatic mutation carriers and non-carriers and Student’s *t*-tests between bvFTD patients and controls. Comparisons of categorical variables were analyzed using the chi-square or Fisher’s exact tests. The structural and ^18^F-FDG PET data were subjected to voxel-wise whole-brain two-sample *t*-tests based on the framework of a general linear model (GLM) in SPM12, with age and sex as covariates. In addition, total intracranial volume was also used as a covariate for structural data analysis. The brain regions with significant volume and FDG changes were determined using a voxel-threshold of *p* < 0.05 (familywise error [FWE]-corrected). The beta-coefficients of the two-sample *t*-test for gray matter volume and FDG metabolism were additionally calculated between carriers and non-carriers, and the effect size threshold was defined by beta > 0.8. For the comparison of network measures, we tested the statistical significance of the differences using non-parametric permutation tests with 5000 permutations based on the following methods: tests were performed by randomly permuting the subjects from both groups and calculating the differences in graph measures between the new randomized groups. This procedure was repeated 5000 times to obtain the distribution of between-group differences. The *p*-values were calculated as the fraction of the difference in distribution values that exceeded the difference value between the actual groups. For all analyses, a *p*-value < 0.05 indicated statistical significance. We performed multiple corrections using the false discovery rate for analyzing local metabolic connectivity changes.

## Results

### Demographic features of the subjects

Demographic, cognitive and behavioral features of the sample are presented in Table 1. There were no significant differences in the estimated years from symptom onset, or MMSE, MoCA, CDR, AVLT, NPI-Q, MBI-C, and FBI scores between asymptomatic subjects with and without the MAPT P301L mutation. The mean (standard deviation) estimated years from the symptom onset was 8.33 (1.875) years in the mutation carrier group, with a range of 4 to 13 years. As shown in Table 1, specific neuropsychological tests and behavioral measures differed significantly between bvFTD patients and controls.

### Gray matter loss

No gray matter loss was identified in asymptomatic MAPT subjects compared with non-carriers based on a voxel-threshold of *p* < 0.05 (FWE-corrected). Subjects with bvFTD showed a widespread pattern of gray matter loss that involved the temporal, frontal, insular, and limbic lobes compared with the controls (Fig. S1A).

The additional post hoc analysis examining the effect size between the carriers and non-carriers with a cutoff value defined as > 0.8 showed gray matter in the inferior temporal gyrus and inferior frontal gyrus (Fig. S2A, Table S1).

### Regional ^18^F-FDG uptake

We first examined the significant differences in ^18^F-FDG uptake between mutation carriers and non-carriers at the voxel level; however, no region survived FWE correction (*p*corr < 0.05). Compared with controls, bvFTD patients had a broader pattern of lowered FDG uptake that involved the temporal lobe, frontal lobe, insular lobe, limbic lobe, anterior cingulate, caudate, and putamen (Fig. S1B).

The additional post hoc analysis examining the effect size between the carriers and non-carriers with a cutoff value defined as > 0.8 revealed decreased metabolism in the inferior frontal gyrus (triangular part) and increased metabolism in the interior frontal gyrus (orbital part), which was shown in Fig. S2B and Table S1.

### Graph theoretical analysis of the metabolic network

#### Global network analysis

For global parameters (Table 2), hierarchy, global efficiency, local efficiency, and clustering coefficients were not significantly different between asymptomatic mutation carriers and non-carriers within the same family. However, global parameters were significantly lower in bvFTD patients than in controls.

**Table 2:** Global graph analysis properties of the brain network

	Asymptomatic MAPT carriers (*n* = 6)	Non-carriers (*n* = 12)	bvFTD patients (*n* = 32)	Controls (*n* = 33)	*p* value*, MAPT carriers vs non-carriers	*p* value*, bvFTD patients vs controls
Assortativity	0.161	0.011	0.114	− 0.003	–	–
Hierarchy	− 0.126	− 0.006	− 0.029	0.031	–	0.026
Cluster coefficient	0.891	0.915	0.881	0.933	–	0.03
Global_Efficiency	0.628	0.618	0.383	0.529	–	0.003
Local_Efficiency	0.670	0.665	0.388	0.561	–	0.003
Synchronization	0.144	0.032	0.248	0.060	–	–

#### Hub regions

**Fig. 1:**
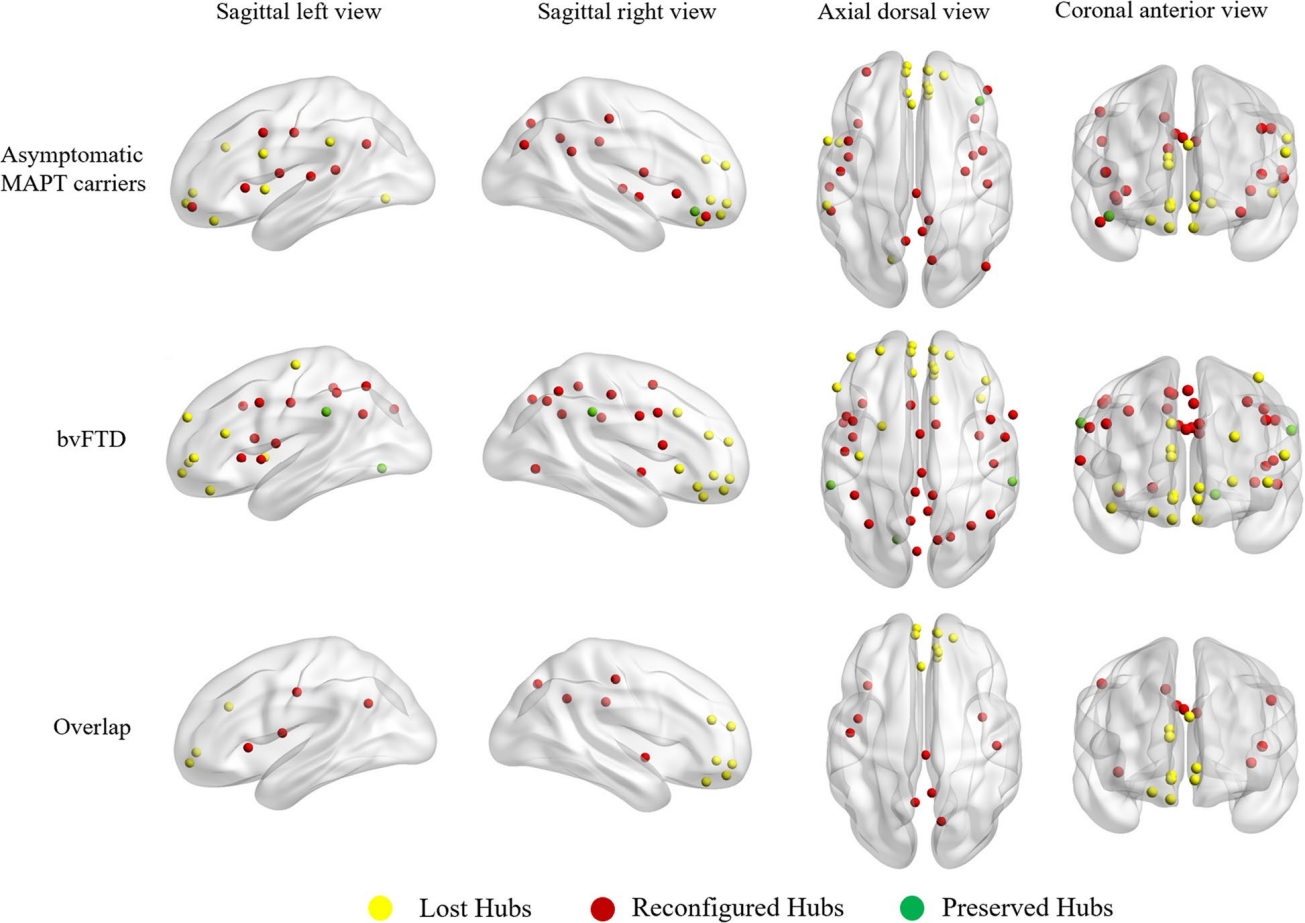
Hub reconfiguration in asymptomatic MAPT carriers and bvFTD patients. The lost and reconfigured hubs, which were consistently present in asymptomatic MAPT carriers and FTD patients, are shown in the overlap. Yellow circles represent the lost hubs (hubs present in controls but not in asymptomatic MAPT carriers or bvFTD patients); red circles represent the reconfigured hubs (nodes in asymptomatic MAPT carriers or bvFTD patients that assume the role of hubs differ to those in controls); green circles indicate the preserved hubs (hubs present in both asymptomatic MAPT carriers and non-carriers or both bvFTD patients and controls). Hubs were identified as brain regions having a nodal degree centrality one standard deviation higher than the network average. Hubs are represented on a 3D brain template using the BrainNet toolbox

Figure 1 shows the hubs in asymptomatic MAPT carriers and bvFTD patients. We identified 15 hubs in asymptomatic MAPT carriers and 22 hubs in bvFTD patients. In asymptomatic carriers compared with non-carriers and bvFTD patients compared with controls, the hubs were classified into three categories: lost, preserved, and reconfigured hubs. Several regions of the medial prefrontal lobe, including the ventromedial prefrontal cortex (vmPFC), orbitofrontal cortex, and anterior cingulate cortex (ACC), were lost hubs in asymptomatic MAPT carriers, which is consistent with those found in bvFTD patients. The precuneus, posterior cingulate, and anterior and middle insula were reconfigured hubs in both asymptomatic MAPT carriers and bvFTD patients.

#### Regional nodal characteristics

**Fig. 2:**
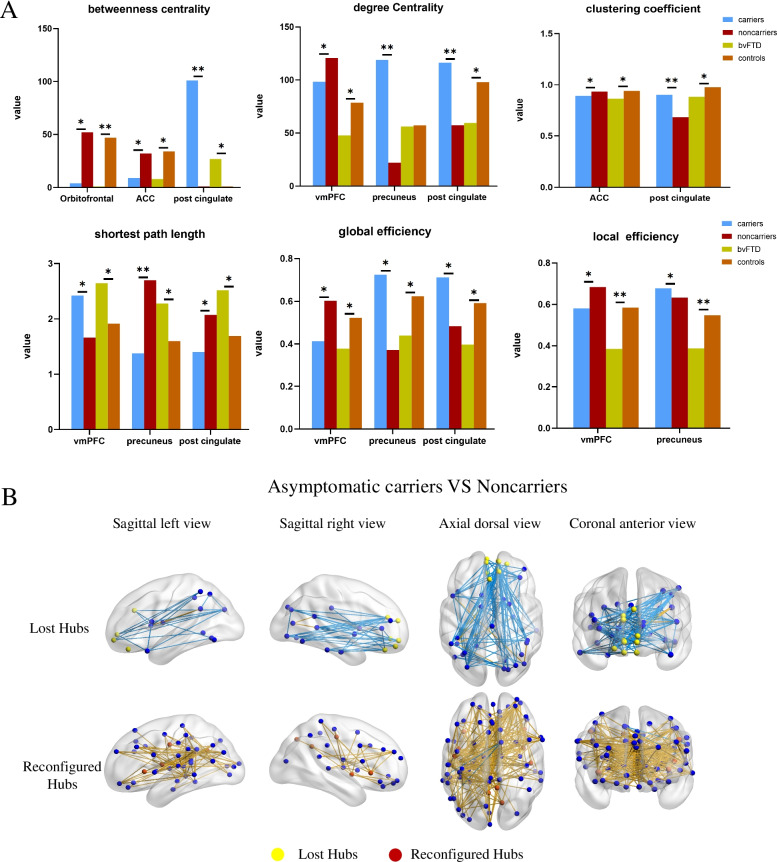
Characteristics of the reorganized hubs. **A** Graph analysis of properties of the nodes that were consistently reorganized in asymptomatic MAPT carriers and FTD patients, including lost and reconfigured hubs. **B** In asymptomatic MAPT carriers, lost hubs had weakened connections with the vlPFC, vPFC, vFC, dlPFC, IPL, IPS, temporal lobe, and occipital lobe, whereas reconfigured hubs had enhanced connections with components of the frontal cortex, including the vmPFC, dlPFC, dFC, vlPFC, aPFC, vFC, orbitofrontal cortex, rectus gyrus, and ACC. Enhanced metabolic connections are represented by yellow lines, and weakened metabolic connections are represented by blue lines. vlPFC, ventrolateral prefrontal cortex; vPFC, ventral prefrontal cortex; vFC, ventral frontal cortex; dlPFC, dorsolateral prefrontal cortex; IPL, inferior parietal lobule; IPS, intraparietal sulcus; vmPFC, ventromedial prefrontal cortex; dFC, dorsal prefrontal cortex; aPFC, anterior prefrontal cortex; ACC, anterior cingulate cortex

At the local level, we focused on the nodes that were consistently reorganized in asymptomatic MAPT carriers and bvFTD patients, including both lost and reconfigured hubs. Significant differences in nodal parameters were found in asymptomatic mutation carriers (Fig. 2A). In terms of degree centrality, global efficiency, local efficiency, and shortest path length, lost hubs (vmPFC, orbitofrontal cortex, and ACC) had lower efficiency in asymptomatic mutation carriers than in non-carriers. Higher efficiency of local information processing in reconfigured hubs (precuneus and posterior cingulate) was found in asymptomatic MAPT carriers than in non-carriers. BvFTD patients showed lower efficiency of local information processing in the above nodes than that of controls.

#### Inter-regional connectivity

**Fig. 3:**
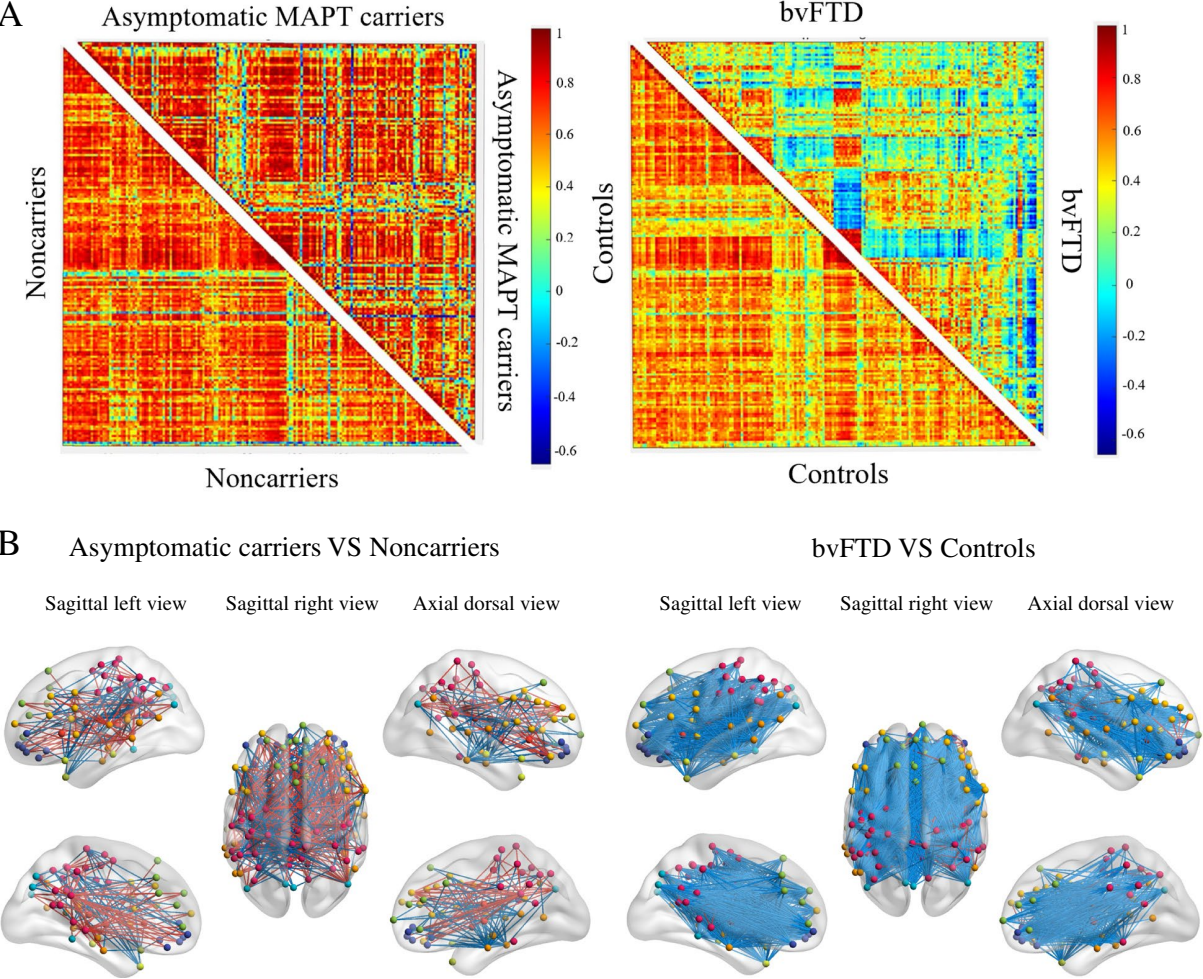
Whole-brain analysis of the network topology and connectome. **A** Whole-brain metabolic connectivity matrices with the red-blue color gradient representing the strength of the correlation between two nodes. Metabolic network topology was disrupted in asymptomatic MAPT carriers and bvFTD patients. **B** Connectomes projected onto a 3D brain template. Global connectivity reconfiguration was present in asymptomatic MAPT carriers and bvFTD patients. Enhanced metabolic connections are represented by red lines, and weakened metabolic connections are represented by blue lines

The metabolic connectivity matrix of both asymptomatic MAPT carriers and bvFTD patients showed a whole-brain reconfiguration (Fig. 3). In asymptomatic MAPT carriers, we focused mainly on the connections associated with lost hubs and reconfigured hubs that were consistently present in asymptomatic MAPT carriers and bvFTD patients, as shown in Fig. 2B. The results showed that asymptomatic mutation carriers had weaker connections of the lost hubs than those of non-carriers. However, the reconfigured hubs had abnormally enhanced connections with components of the frontal cortex. In bvFTD patients, weakened connections were observed in widespread brain regions. Abnormally enhanced connections were concentrated in only the precuneus and posterior cingulate. Significant changes in metabolic connectivity are shown in Fig. 3.

### Default mode network and salience network

**Fig. 4:**
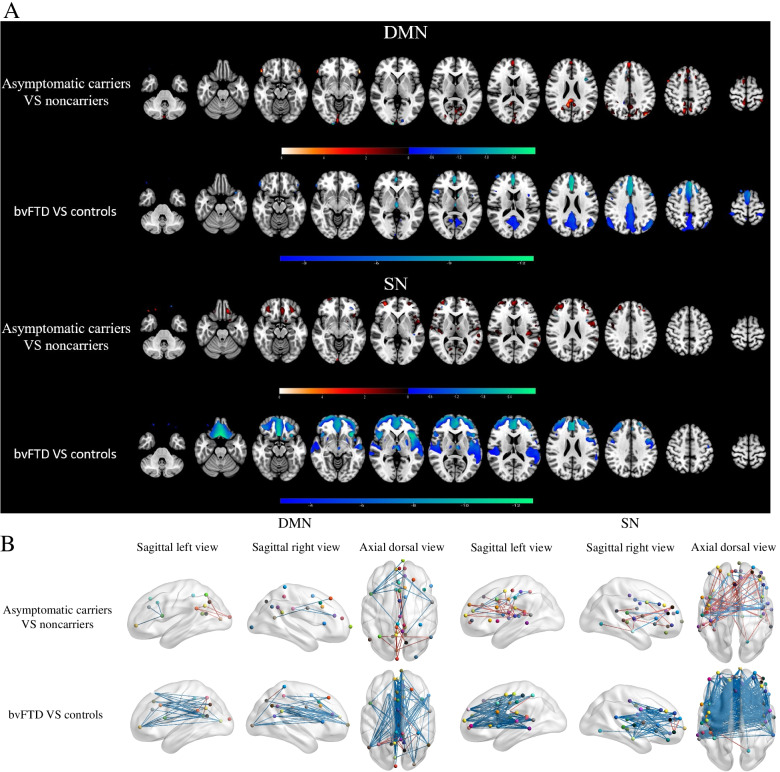
Metabolic connectivity of the DMN and SN. **A** ICA estimates of the difference at the voxel level in the DMN and SN network between MAPT carriers and non-carriers (uncorrected *p* < 0.005) and between bvFTD patients and controls (FWE *p* < 0.05). The significant difference between subjects with MAPT carriers > non-carriers is shown in red. Blue highlights the differences for subjects with MAPT carriers < non-carriers and bvFTD patients < controls. **B** Differences in connectivity within the DMN and SN in MAPT carriers compared with non-carriers and bvFTD patients compared with controls using non-parametric permutation tests with 5000 permutations (*p* < 0.05), which are represented on a 3D brain template using the BrainNet toolbox. Increased metabolic connections are represented by red lines, decreased metabolic connections by blue lines

The default mode network (DMN) and salience network (SN) were highlighted because they had lost hubs (i.e., the vmPFC and ACC, respectively) as one of their central nodes. Alterations in the metabolic connectivity of the DMN and SN were present in both asymptomatic MAPT carriers and bvFTD patients, as shown in Fig. 4. For the SN, there was enhanced connectivity in asymptomatic mutation carriers. For the DMN, we found increased connectivity in the precuneus and thalamus, and small regions of reduced connectivity were identified in the prefrontal cortex in asymptomatic carriers. In contrast, bvFTD patients showed widespread hypoconnectivity within the DMN and SN.

## Discussion

This study is the first to provide evidence that the topological organization of the metabolic brain network is altered in asymptomatic MAPT P301L subjects. Furthermore, asymptomatic MAPT P301L carriers have early involvement of medial prefrontal regions (vmPFC, orbitofrontal cortex, and ACC) and abnormally increased connectivity in task-related regions (DMN and SN), which may be a compensatory response to maintain the global efficiency of the brain network.

We detected aberrations of network topology and metabolic connectivity in asymptomatic MAPT carriers, which has not been specifically investigated before. Moreover, the alteration of the metabolic network topology in asymptomatic MAPT P301L mutation carriers was overlapped with those observed in bvFTD. These results revealed that disorders of neuronal connections were present in the presymptomatic stage of genetic FTD, which was in line with the previous study [6, 20]. The atrophy and hypometabolism of the anterior cingulate cortex failed to be detected in our asymptomatic carriers, which was inconsistent with a previous study with six MAPT P301L mutation carriers from five families [7]. The small sample size and genetic heterogeneity may contribute to this discrepancy. Notably, the anterior cingulate cortex was identified as the lost hub and decreased connectivity in our study, suggesting its involvement in asymptomatic MAPT mutation carriers. Regardless, our findings suggest that changes in the brain metabolic network are an early feature of genetic FTD. Additionally, applying the hybrid PET/MRI system allowed us to match anatomical and functional modalities in the same individual, which provided a comprehensive illustration of the subtle brain changes in asymptomatic mutation carriers. Moreover, a strength of our study is that we selected a large family to explore the effects of MAPT mutation in asymptomatic carriers compared with non-carriers, which minimizes the effects of interfamilial genetic variation on brain morphology and network organization [21]. Taken together, these findings indicate that the topological properties and connectomics of metabolism networks may be a particularly sensitive and useful method for investigating and monitoring the earliest stages of FTD in individuals with this underlying genetic basis.

Our graph theory results identified lost hubs in the vmPFC, orbitofrontal cortex, and ACC and reconfigured hubs in the precuneus, posterior cingulate, and insula in asymptomatic mutation carriers. It is worth noting that the reduced connectivity observed was consistent with hub loss. The pattern of hub reorganization and reduced connectivity during the asymptomatic stage shows a large resemblance to the regions observed in sporadic bvFTD patients. Furthermore, the involvement of these regions corresponds with the pattern of neuropathology in FTD, in which the initial changes occur in the medial prefrontal cortex (mPFC), while the parietal lobe remains preserved [22]. Accordingly, alterations in the personality and behaviors of bvFTD patients, such as emotional blunting, loss of empathy, and an inability to consider the thoughts and perspectives of others, arise because of the early degeneration of the mPFC [23, 24]. Functional network alterations have been reported to follow a topological distribution, which suggests stepwise spreading [25]. Reduced connectivity was evident only in the medial prefrontal regions in asymptomatic carriers but was apparent also in other brain regions in FTD patients, which indicates that the mPFC is implicated early in tau-mediated FTD. Correspondingly, this pattern of involvement is also in line with the characteristics of tau deposition identified in vivo using ^18^F-flortaucipir in asymptomatic MAPT P301L carriers [26]. Above all, our findings suggest that the abnormalities of functional brain networks in MAPT carriers start in the medial prefrontal lobe during the pre-symptomatic phase and eventually spread into the other regions.

Furthermore, lesion effects are not always limited to circumscribed locations because of diaschisis affecting remotely connected sites; therefore, a resting-state network-based approach is needed to gain a better understanding of the structure-function relationships in the mPFC [27]. We focused on specific vmPFC-linked networks (DMN) and ACC-linked networks (SN). Consistent with recently reported fMRI findings in asymptomatic granulin (GRN) carriers [28, 29], our results revealed selectively increased connectivity within the DMN and SN during the presymptomatic stage but a reduction in FTD patients. The DMN and SN are involved in functions such as emotional processing and mentalizing/social cognition, which likely contribute to various critical features of FTD [6, 23]. Increased task dependency of the DMN and SN connectivity profiles in asymptomatic MAPT carriers may represent a compensatory mechanism of brain plasticity in the presence of pathology that selectively targets neurons in the medial prefrontal areas. Although our results contrast with a previous study that found reduced DMN connectivity in asymptomatic MAPT carriers, we believe our findings are likely a feature of primary tauopathy [6]. Discordance across studies may be attributed to the differences in disease-related stage and genetic subtypes of MAPT. Taken together, we speculate that the enhancement in the DMN and SN compensates for mPFC deficits by reconfiguration during the early stages of FTD, which contributes to the maintenance of efficient information transfer.

Accordingly, asymptomatic mutation carriers in our study showed normal global properties of brain network topology, which included global efficiency, hierarchy, local efficiency, and clustering coefficients, whereas bvFTD patients exhibited a breakdown of these global parameters. This suggests that mutation carriers maintain the information exchange efficiency of the overall brain network before the onset of symptoms, which may contribute to the preservation of cognition. Consistent with our findings, previous studies have revealed that global efficiency, which measures the ability of a network to transmit information at the global level, is associated with the degree of cognitive impairment [30, 31]. Moreover, normal network efficiency and increased connectivity have been observed during prodromal phases, followed by decreases in global efficiency and local connectivity in symptomatic phases due to the dissipation of neural compensation [32]. Two previous fMRI studies indicated that abnormally increased connectivity supports cognitive health in asymptomatic GRN carriers, whereas the specific pattern of network reorganization, especially those modulated by different genetic defects, remains elusive [28, 29]. In our study, normal network efficiency may be attributed to the increased connectivity in the DMN and SN to compensate for the mPFC deficit. Furthermore, topology reconfiguration of the metabolic network may maintain efficient information processing, which may support cognitive well-being in MAPT mutation carriers before the onset of symptoms. In future studies, the distinct pattern of metabolic network reorganization requires confirmation in a larger cohort with different genetic profiles.

## Limitations

This study has several limitations. First, the results were limited by the small sample size because FTD families with the MAPT mutation are rare. Second, this was a cross-sectional study. Future longitudinal studies with postmortem confirmation are essential to capture the complexity and progression of metabolic network topology and connectivity alterations observed in asymptomatic MAPT mutation carriers. Third, the asymptomatic MAPT subjects and subjects with bvFTD were each matched to a control group, which reduces their comparability. Nevertheless, the groups were as close as possible in age; moreover, age was included as a covariate in all analyses to further minimize potential confounds.

## Conclusions

Our findings suggested that asymptomatic MAPT mutation carriers have alterations in the metabolic network topology. Before the age of symptom onset in genetic FTD, abnormal local topological properties and hypometabolic connectivity were present in medial prefrontal areas (vmPFC, orbitofrontal cortex, and ACC), and the functional connections of the task-related regions, which included the DMN and SN, were reconfigured to compensate for the mPFC deficit to maintain efficient network topology and information processing for preserving cognitive function. Topological properties and metabolic connectivity may be valuable for future investigations and the monitoring of the earliest stages of FTD in individuals with an underlying genetic basis.

## Supplementary Information

**Additional file 1: Figure S1.** Regions of atrophy and hypometabolism. Reduced gray matter volume and glucose metabolism are depicted in blue. Data were analyzed at a height threshold of *p* < 0.001 and were cluster-level corrected for FWE at *p* < 0.05. (A) Decline in gray matter volume in MAPT mutation carriers vs non-carriers and bvFTD patients vs controls. (B) Projections of areas with relative hypometabolism in MAPT carriers compared with non-carriers and bvFTD patients compared with controls.

**Additional file 2: Figure S2.** Regions of atrophy and hypometabolism in MAPT mutation carriers vs non-carriers. This is additional post-hoc analysis examining the effect size between the two groups with cut-off value defined as >0.8. Decline in gray matter volume of inferior temporal gyrus and inferior frontal gyrus (Triangular part) in MAPT mutation carriers vs non-carriers. Projections of areas with relative hypometabolism of inferior frontal gyrus (Triangular part), and hypermetabolism in interior frontal gyrus (Orbital part) in MAPT carriers compared with non-carriers. Reduced gray matter volume and glucose metabolism are depicted in blue. Increased glucose metabolism is depicted in red.

**Additional file 3: Table S1.** Spatial coordinates and peak values of brain areas showing significant GM density and metabolism changes between asymptomatic MAPT carriers and non-carriers.

## Data Availability

The datasets used and analyzed during the current study are available from the corresponding author on reasonable request.

## Abbreviations

ACC: Anterior cingulate cortex
DMN: Default mode network
FTD: Genetic frontotemporal dementia
MAPT: Microtubule-associated protein tau
SN: Salience network
vmPFC: Ventromedial prefrontal cortex
