# Supplementary figures and images for: Reconfigured metabolism brain network in asymptomatic microtubule-associated protein tau mutation carriers: a graph theoretical analysis
Source: Alzheimers Res Ther. 2022 Apr 11;14:52. doi: 10.1186/s13195-022-01000-z (PMC8996677; doi:10.1186/s13195-022-01000-z)

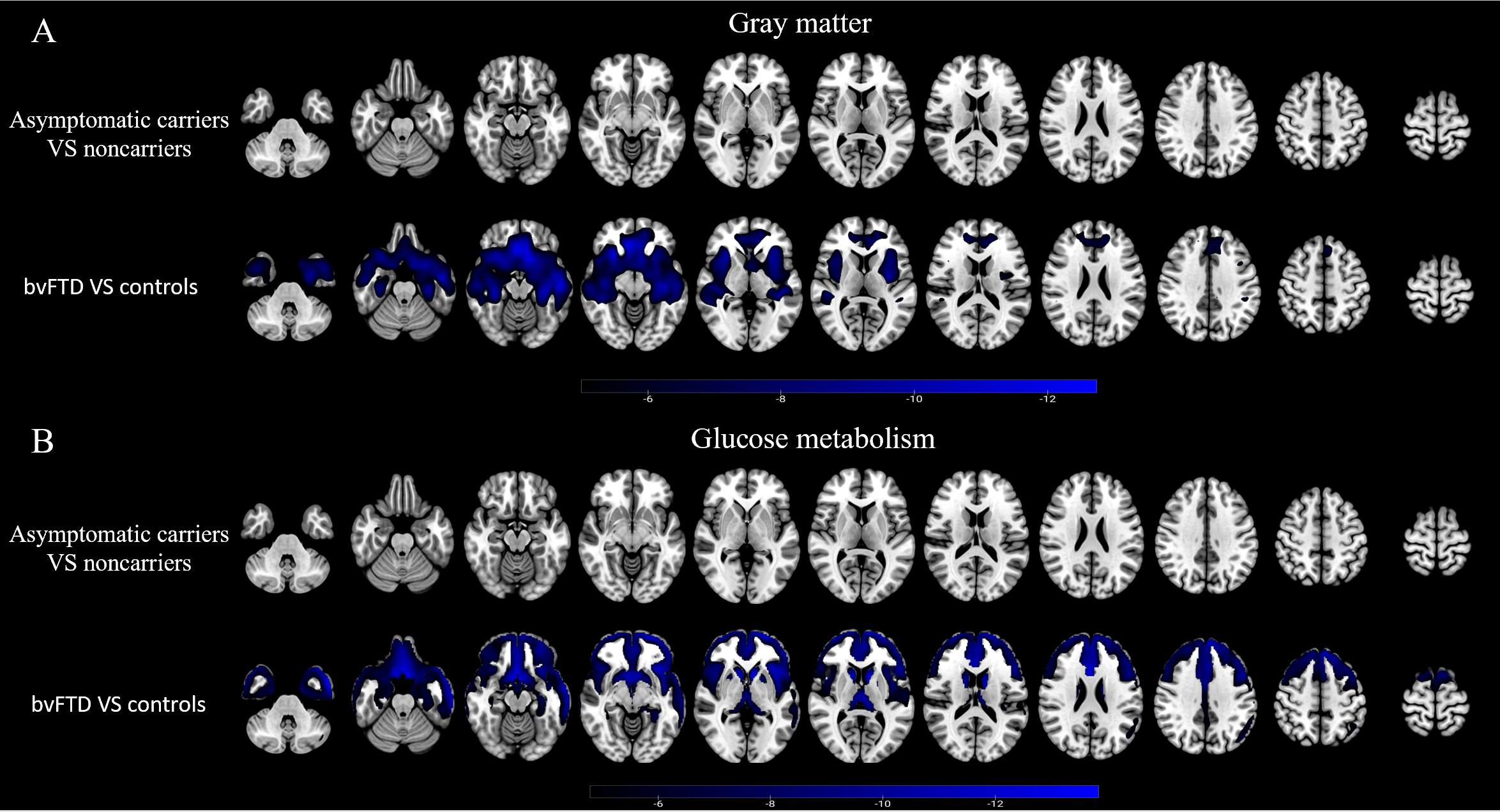

Supplement: Supplementary file 1 — Additional file 1: Figure S1. Regions of atrophy and hypometabolism. Reduced gray matter volume and glucose metabolism are depicted in blue. Data were analyzed at a height threshold of p < 0.001 and were cluster-level corrected for FWE at p < 0.05. (A) Decline in gray matter volume in MAPT mutation carriers vs non-carriers and bvFTD patients vs controls. (B) Projections of areas with relative hypometabolism in MAPT carriers compared with non-carriers and bvFTD patients compared with controls. [file 13195_2022_1000_MOESM1_ESM.png]

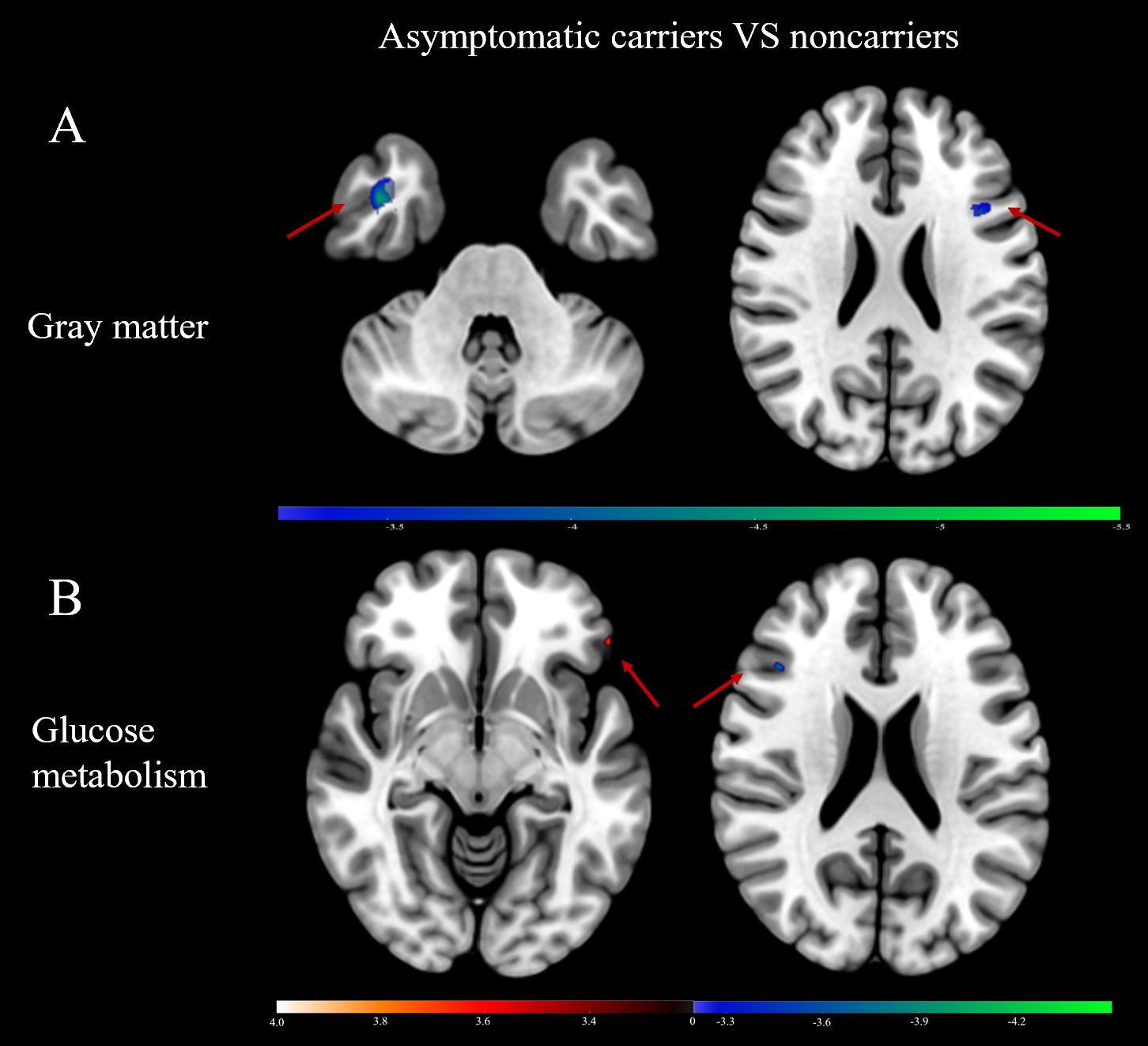

Supplement: Supplementary file 2 — Additional file 2: Figure S2. Regions of atrophy and hypometabolism in MAPT mutation carriers vs non-carriers. This is additional post-hoc analysis examining the effect size between the two groups with cut-off value defined as >0.8. Decline in gray matter volume of inferior temporal gyrus and inferior frontal gyrus (Triangular part) in MAPT mutation carriers vs non-carriers. Projections of areas with relative hypometabolism of inferior frontal gyrus (Triangular part), and hypermetabolism in interior frontal gyrus (Orbital part) in MAPT carriers compared with non-carriers. Reduced gray matter volume and glucose metabolism are depicted in blue. Increased glucose metabolism is depicted in red. [file 13195_2022_1000_MOESM2_ESM.png]
