# Supplementary material for: Reconfigured metabolism brain network in asymptomatic microtubule-associated protein tau mutation carriers: a graph theoretical analysis
Source: Alzheimers Res Ther. 2022 Apr 11;14:52. doi: 10.1186/s13195-022-01000-z (PMC8996677; doi:10.1186/s13195-022-01000-z)
Supplement: Supplementary file 3 — Additional file 3: Table S1. Spatial coordinates and peak values of brain areas showing significant GM density and metabolism changes between asymptomatic MAPT carriers and non-carriers. [file 13195_2022_1000_MOESM3_ESM.docx]

Supplemental Table 1 Spatial coordinates and peak values of brain areas showing significant GM density and metabolism changes between asymptomatic MAPT carriers and noncarriers.

| Region | Anatomical localization | Side | Cluster Size | MNI coordinate | Effect  size |
| --- | --- | --- | --- | --- | --- |
| **GM atrophy** | | | | | |
| Temporal lobe | Inferior temporal gyrus | R | 358 | 41 2 -38 | 1.32 |
| Frontal lobe | Inferior frontal gyrus  (triangular part) | L | 88 | -41 14 24 | 0.91 |
| **hypermetabolism** |  |  |  |  |  |
| Frontal Lobe | Interior frontal gyrus (orbital part) | L | 70 | -54 34 -8 | 0.8 |
| **hypometabolism** | | | | | |
| Frontal lobe | Inferior frontal gyrus (triangular part) | R | 97 | 42 25 21 | 0.96 |
